# Vaccination with *Plasmodium knowlesi* AMA1 Formulated in the Novel Adjuvant Co-Vaccine HT™ Protects against Blood-Stage Challenge in Rhesus Macaques

**DOI:** 10.1371/journal.pone.0020547

**Published:** 2011-05-31

**Authors:** Muzamil Mahdi Abdel Hamid, Edmond J. Remarque, Leonie M. van Duivenvoorde, Nicole van der Werff, Vanessa Walraven, Bart W. Faber, Clemens H. M. Kocken, Alan W. Thomas

**Affiliations:** Department of Parasitology, Biomedical Primate Research Centre, Rijswijk, The Netherlands; Queensland Institute of Medical Research, Australia

## Abstract

*Plasmodium falciparum* apical membrane antigen 1 (PfAMA1) is a leading blood stage vaccine candidate. *Plasmodium knowlesi* AMA1 (PkAMA1) was produced and purified using similar methodology as for clinical grade PfAMA1 yielding a pure, conformational intact protein. Combined with the adjuvant CoVaccine HT™, PkAMA1 was found to be highly immunogenic in rabbits and the efficacy of the PkAMA1 was subsequently tested in a rhesus macaque blood-stage challenge model. Six rhesus monkeys were vaccinated with PkAMA1 and a control group of 6 were vaccinated with PfAMA1. A total of 50 µg AMA1 was administered intramuscularly three times at 4 week intervals. One of six rhesus monkeys vaccinated with PkAMA1 was able to control parasitaemia, upon blood stage challenge with *P. knowlesi* H-strain. Four out of the remaining five showed a delay in parasite onset that correlated with functional antibody titres. In the PfAMA1 vaccinated control group, five out of six animals had to be treated with antimalarials 8 days after challenge; one animal did not become patent during the challenge period. Following a rest period, animals were boosted and challenged again. Four of the six rhesus monkeys vaccinated with PkAMA1 were able to control the parasitaemia, one had a delayed onset of parasitaemia and one animal was not protected, while all control animals required treatment. To confirm that the control of parasitaemia was AMA1-related, animals were allowed to recover, boosted and re-challenged with *P. knowlesi* Nuri strain. All control animals had to be treated with antimalarials by day 8, while five out of six PkAMA1 vaccinated animals were able to control parasitaemia. This study shows that: i) Yeast-expressed PkAMA1 can protect against blood stage challenge; ii) Functional antibody levels as measured by GIA correlated inversely with the day of onset and iii) GIA IC_50_ values correlated with estimated *in vivo* growth rates.

## Introduction

The *Plasmodium falciparum* parasite is responsible for at least 300 million cases of malaria [1] and about 800,000 deaths every year [2]. The majority of these deaths occur in children under five and nulliparous women in sub-Saharan Africa [3]. An effective malaria vaccine combined with existing anti-malarial strategies (e.g. indoor residual spraying, bed-nets, antimalarial drugs etc.) would certainly help to reduce malaria-related morbidity and mortality, and as such would be a much needed addition to the arsenal.

The leading vaccine candidate Apical Membrane Antigen 1 (AMA1) was first discovered as a minor invariant merozoite antigen in *Plasmodium knowlesi* [4], [5] able to protect rhesus monkeys from asexual blood stage challenge with *P. knowlesi* (Pk) [6]. The protective effect of AMA1 vaccination has since been demonstrated in mouse and simian malaria models [7]–[9] and has spurred the clinical development of *P. falciparum* AMA1 [7].

*P. knowlesi* causes a fulminent infection in rhesus monkeys, often resulting in high parasitaemias similar to levels observed in *P. falciparum* infections in humans, and ultimately in death if left untreated [10]–[12]. *P. knowlesi* has recently also been shown to infect humans under natural conditions and was shown to be a contributing cause of death [13], [14].

The clinical development of AMA1 has resulted in several trials [15]–[19], including a phase IIa mosquito challenge of non-malaria exposed volunteers [20] and a phase IIb trials in Malian children [21], [22]. The phase IIa study did show a small, but significant, delay in the pre-patent phase as determined by PCR data modelling, whilst time to patency was similar in vaccinees and controls, despite high Growth Inhibition Assay (GIA) titres in the vaccinees [20]. One phase IIb study in Malian children using Alum did not show any significant protection [22], whereas a Phase IIb study using a potent adjuvant (AS02) revealed a modest non-significant vaccine efficacy of about 17% when analysed against all AMA1 alleles in infected subjects. The number of parasites expressing the homologous 3D7-AMA1 was too low allow for efficacy to be determined, but when data were analysed for parasites expressing the homologous 3D7-AMA1 haplotype in the highly polymorphic C1 region, vaccine efficacy was estimated at 64% [23]. This has fuelled the debate whether vaccination with AMA1 would have protective potential in humans.

The problem in pre-clinical and early clinical evaluation of a vaccine is the lack of correlates of protection. This hampers vaccine development, as without these correlates it is not clear what kind of responses a vaccine needs to induce in order to protect. A recent paper by Dutta et al. suggests that high titres of, functionally active, antibodies against AMA1 are required for protection in a non-human primate (*Aotus*) model [8]. We use non-human primate models to establish correlates of protection, as an alternative to rodent malaria models, that may be less representative for the human malaria's [24]. The Pk rhesus monkey model allows proof of concept testing of *P. falciparum* and *P. vivax* vaccine candidates for which Pk orthologs exist as well as evaluation of vaccines against *P. knowlesi*. Humoral and cellular immune responses can be evaluated in depth. Parasite challenge can be performed either by infected mosquito bite, sporozoite injection (i.v., s.c. or i.d.) or i.v. inoculation of infected red blood cells, and followed-up beyond patency, allowing for a larger window of opportunity to detect vaccine effects as compared to human phase IIa trials. Moreover, rhesus monkeys can be infected repeatedly with *P. knowlesi* before immunity develops [11]. Unlike clinical trials, trials in monkeys do not require cGMP-grade materials (although at least GLP grade antigen is preferred), and formal pharmacotoxicology testing is not mandatory. In addition, preliminary safety profile of vaccines can be established in monkeys (behaviour, appetite, local reactions, clinical chemistry and haematology).

In this study, we used *P. knowlesi* in *Macaca mulatta* (rhesus monkey) as a model to test the efficacy of the blood stage vaccine candidate AMA1. The vaccine antigen PkAMA1 was expressed in the methylotrophic yeast *Pichia pastoris*, purified and quality controlled similar to the cGMP production of PfAMA1 [25]. The antigen used in the control group, cGMP-grade PfAMA1 [25], induces PkAMA1 cross-reactive antibodies detectable in ELISA, but results obtained in our laboratory have shown that these antibodies do not have appreciable GIA activity when tested on *P. knowlesi*.

A number of clinical studies, among others the one describing a phase Ia clinical study, showed that the adjuvant Alhydrogel® is not potent enough to achieve high antibody levels for AMA1 resulting in high GIA titres [15], [16], [22]. Therefore we used the novel adjuvant, CoVaccine HT™ that induces significantly higher levels of functional antibodies to AMA1 than a proprietary water in oil adjuvant, whilst being well tolerated by pigs and macaques [26], [27]. The active ingredients of CoVaccine HT™ are sucrose fatty acid sulphate esters (SFASE) and an oil-in-water (squalane and Polysorbate-80) emulsion [26], [28]. It has been shown that this adjuvant enhances both humoral and cell mediated responses when used with subunit vaccines [26]. CoVaccine HT™is currently in clinical development (http://www.clinicaltrials.gov/ct2/results?term=CoVaccineht).

Here we report the parasitological and immunological results of a rhesus macaque vaccination – blood-stage challenge study with *Pichia pastoris* expressed PkAMA1 formulated with CoVaccine HT™. We show that there is a correlation between functional (GIA) antibody levels and the delay of onset of parasitaemia as well as with *in vivo* parasite multiplication rates. The promising results obtained here warrant clinical development of CoVaccine HT™ adjuvanted *P. falciparum* and, possibly, *P. knowlesi* AMA1 as vaccines for life-threatening human *Plasmodium* infections.

## Materials and Methods

### Cloning of *Plasmodium knowlesi ama1*

A synthetic gene, codon optimised for expression in *Pichia pastoris*, comprising domain I-II-III of *Plasmodium knowlesi* H strain AMA1 (amino acids 43 to 487) (Genbank accession number XM_002259303), was constructed by DNA2.0 (Menlo Park, CA). Five Potential N-glycosylation sites were removed, by replacing one of the amino acid in the NX[T/S] fingerprint with an amino acid present in an AMA1 orthologue, analogous to what was done for *P. falciparum* AMA1 [29]. Asn_107_ was replaced by Lys, Ser_178_ was replaced by Asn, Asn_190_ was replaced by Glu, Ser_240_ was replaced by Arg and Asn_441_ was replaced by Gln. A PCR forward primer (PkF: 5′GGAATTCCCAATCATTGAGAGATC3′) with a 5′ *Eco*R1 restriction site and two reverse primers with 3′*Xba*I restriction sites (PkRIII: 5′CGTCTAGACCTTCCTAGTAACATTTTCTTC3′; PkRII: 5′GCTCTAGACCTGGAAACTCGTTGTCTAC 3′) were used to amplify PkAMA1 domain I-II-III (aa 43–487) and PkAMA1 domain I-II (aa 43–387), respectively. PCR products were cloned into *Eco*RI-*Xba*I sites of the pPicZαA vector (Invitrogen, Leek, The Netherlands) in frame with the vector-encoded myc-epitope and hexa-Histag. After transformation of *E. coli* DH5α cells, plasmids were isolated, the sequence was confirmed by DNA-sequencing (Baseclear, Leiden, The Netherlands), and used to transform *P. pastoris* KM71H following the manufacturer's protocols.

### Selection of *Pichia* clones

Transfected *P. pastoris* were tested for protein production. Cells were cultured in 10 mL glycerol-containing medium for 24–30 h (30°C, 200 rpm). Cells were harvested by centrifugation and resuspended in 4 mL methanol-containing medium, and then cultured for 24 h. After low-speed centrifugation, the culture supernatant was harvested and 20 µL of the culture supernatant was analyzed by non-reducing SDS-PAGE with coomassie brilliant blue staining or Western blotting with the reduction sensitive monoclonal antibody R3/1C2 [4], which also served to confirm the identity of the proteins.

### Fermentation of *Pichia pastoris*

Fermentation runs for either construct were done in a 7 L fermentor (Applikon, Schiedam, The Netherlands) with a starting volume of 2 L. Fermentations were essentially performed according to the instructions provided by the manufacturer of the yeast strain and the vector (Invitrogen, Leek, The Netherlands) for *P. pastoris* Mut^S^ cells [25].

### Purification of recombinant PkAMA1 DI-II-III and PkAMA1 DI-II proteins

The methodology used for the production of PkAMA1 proteins was similar to what was used for PfAMA1 [25]. Proteins were purified using two chromatography purification steps, Nickel IMAC affinity chromatography followed by size exclusion chromatography. In brief, filtered culture supernatant was loaded on a Ni-IMAC-Sepharose column (GE Healthcare, Diegem, Belgium). Washing was done with 10 column volumes of 20 mM Na-phosphate, pH 7.8, 500 mM NaCl and 10 mM imidazole to remove contaminating proteins. PkAMA1 proteins were eluted with 20 mM Na-phosphate pH 7.8, 500 mM NaCl and 100 mM imidazole. This fraction was exchanged into PBS using ultrafiltration concentrator tubes (10 kDa cutt-off; Millipore, Amsterdam, The Netherlands). Protein fractions from different fermentations were pooled and concentrated to 1 mL. Subsequently this fraction was injected on a Superdex 75 preparative size exclusion chromatography column (GE healthcare, Diegem, Belgium). Fractions containing PkAMA1 proteins of 50 kDa (PkAMA1 D I-II-III) or 40 kDa (PkAMA1 DI-II) were pooled, concentrated and filter sterilised and then stored at −20°C.

### SDS-PAGE

SDS-PAGE (NuPAGE Novex; Invitrogen, Leek, The Netherlands) 4–12% gradient gels were used with MES-buffer according to the manufacturer's instructions. For densitometry coomassie blue stained gels were scanned with a GS-800 densitometer (Bio-Rad) and analyzed with Quantity one software package version 4.5.

### Conformational integrity assay for PkAMA1

Quantitative binding of PkAMA1 to the conformation-dependent MAb [4] was determined as follows: MAb R3/1C2 (2 mg mL^−1^) was coupled to 1 mL cyanogen bromide (CNBr) activated Sepharose 4 Fast Flow according to the manufacturer's instructions (Amersham Biosciences). Two-hundred µg of PkAMA1 DI-II-III, PkAMA1 DI-II and BSA were diluted in 3 volumes binding buffer (ImmunoPure A, Pierce, Rockford, IL). Samples were separately passed over the immobilized R3/1C2 affinity column and the flow through was collected, and samples containing AMA1 were confirmed to be negative for protein by μBCA protein determination. After extensive washing with 10 bed volumes PBS, bound proteins were eluted with 5–10 volumes 4.0 M NaSCN, pH 7.0. The eluted proteins were exchanged into PBS and concentrated using ultrafiltration concentrator tubes (10 kDa cut off; Millipore, Millipore, The Netherlands). The protein contents in the eluate, flow-through and wash fractions were estimated by μBCA.

### Preparation of AMA1 vaccine formulations

PkAMA1 CoVaccine HT™ (a novel proprietary vaccine adjuvant of Protherics Medicines Development Limited, a BTG Company, London, UK) formulations were prepared in a laminar flow cabinet by mixing and gentle agitation of equal volumes of antigen (200 µg mL^−1^ in PBS, pH 7.4) and CoVaccine HT™ adjuvant (40 mg mL^−1^ of Sucrose Fatty Acid Sulphate Esters [SFASE] in a squalane o/w emulsion) in a glass vial. A 500 µL vaccine dose contained 50 µg of antigen and 10 mg of SFASE. Lyophilised cGMP PfAMA1 [4] was reconstituted with saline for injection to a concentration of 200 µg mL^−1^ and then formulated as above.

### Ethics statement

For the rabbit immunisations, all animals were handled in strict accordance with good animal practice as defined by the Belgian national animal welfare regulations, and all animal work was approved by the ethics committee of the Centre d'Economie Rurale (CER Groupe, Marloie, Belgium). All rabbit handlings were performed at Eurogentec SA, Seraing, Belgium, under permit number LA 1800104.

All rhesus monkeys used in this study were captive bred for research purposes. BPRC housing and animal care procedures are in compliance with Dutch law on animal experiments, European directive 86/609/EEC, and with the “Standard for Humane Care and Use of Laboratory Animals by Foreign Institutions”, identification number A5539-01, provided by the Department of Health and Human Services of the US National Institutes of Health (NIH). The BPRC is compliant with the recommendations of the Weatherall report “The use of non-human primates in research” (http://www.acmedsci.ac.uk/images/project/nhpdownl.pdf).The independent ethics committee at BPRC, constituted according to Dutch law on animal experiments, approved the study protocol (number DEC 552) prior to start of the experiment. In order to minimise suffering, venapunctures, immunisations and passage of blood stage parasites, were performed on ketamine-sedated monkeys. Animals were treated with antimalarials when the parasitaemia following challenge exceeded 2%. Every day animals were carefully observed for their general health (behaviour, alertness, appetite and stool). In the event abnormalities were observed, which did not happen during the course of this study, a veterinarian was available at all times to take appropriate action. During the course of the study all animals received a variety of enrichment items (e.g. toys, food puzzles).

### Rabbit immunisations

Rabbit immunisations were conducted at Eurogentec, Seraing, Belgium under national animal welfare regulations. Two groups of rabbits (N = 5) were injected intramuscularly with 0.5 mL vaccine containing 50 µg of purified PkAMA1 protein formulated in CoVaccine HT™. Two booster injections of 50 µg were given at days 28 and 56 after the primary dose. Bleeds were taken from each animal before immunisation as well as two weeks after the second immunisation. Exsanguination was done two weeks following the last immunisation (day 70).

### Vaccination, boost, and blood sampling of rhesus macaque monkeys

**Figure 1:**
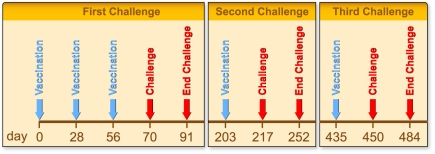
Study schedule. Timing of immunisations and challenges.

12 healthy adult rhesus macaques weighing between 5 to 13 kg, were selected for the study. The animals were all shown to be negative for *Plasmodium* parasites, anti-PfAMA1 or anti-PkAMA1 antibodies. Animals were stratified over two groups of six animals, with similar characteristics with respect to sex, age, and weight. Treatments were then randomly assigned (Table 1). All vaccinations and venapunctures were performed on ketamine-sedated animals. Monkeys in the experimental group (N = 6) were immunised with 50 µg PkAMA1 DI-II-III (aa43–487) formulated in CoVaccine HT™ and the control monkeys (N = 6) were immunised with 50 µg GMP-grade PfAMA1 FVO strain DI-II-III (aa25–545) formulated in CoVaccine HT™. Vaccines were given intramuscularly in alternating upper legs on days 0, 28 and 56. Animals were bled before each immunisation and on days 1, 7, and 14 following each vaccination. After the primary parasite challenge animals were left to recover before a 50 µg booster dose of Pk or PfAMA1 was given on day 203 and animals were re-challenged two weeks later (day 217). The follow up was 35 days. Monkeys were left to recover from the second challenge and were boosted with 50 µg Pk or PfAMA1 on day 435, and challenged again on day 450. Follow up time was 34 days (Figure 1).

**Table 1:** Characteristics of rhesus monkeys at enrolment (Range).

	Treatment	
Characteristic	PkAMA1	PfAMA1
Gender (M ∶ F)	4 ∶ 2	3 ∶ 3
Weight (kg)	8.3 (4.8 to 9.7)	8.4 (5.4 to 9.1)
Age (years)	7.3 (5.2 to 11.4)	7.2 (4.8 to 9.7)

### Inspection of injection sites

Injection sites were inspected on days 0, 1, 7 and 14 following each vaccination. Local reactions for oedema and erythema were scored using the Draize scale [30]. A similar scoring system was applied for the rating of induration and lymph node swelling.

### Clinical chemistry and haematology

Standard parameters were determined for haematology (Sysmex XT 2000iV platform; Goffin Meyvis, Etten-Leur, the Netherlands) and clinical chemistry (Cobas Integra 400; Roche Diagnostics, Basel, Switzerland) on freshly obtained blood samples. The following biochemical measurements were performed on all blood samples taken on days 0, 1, 7 and 14 following each vaccination: alkaline phosphatase, alanine transaminase, aspartate transaminase, gamma glutamyl transpeptidase, bilirubin, cholesterol, lactate dehydrogenase), urea, creatinine, albumin, total protein, glucose, iron, calcium, sodium, potassium, chloride, phosphate and HCO_3_^−^. The following haematological measurements were performed: erythrocyte count, haemoglobin, haematocrit, mean corpuscular volume), mean corpuscular haemoglobin, mean corpuscular haemoglobin concentration, white blood cell count including leukocyte differentiation, platelet count and mean platelet volume. The data were compared with reference values obtained from healthy naive rhesus monkeys. Commercial kits were used according to manufacturer's instruction.

### Blood stage parasites and challenge infection of rhesus macaques

A parasite donor monkey negative for AMA1 antibody and without previous exposure to malaria (also negative for Herpes-B, Simian Retrovirus and STLV) was selected. Cryopreserved wild-type *P. knowlesi* H strain was thawed according to the method described by Trager et al. [31]. The parasite pellet (containing mainly surviving rings) was allowed to recover in gassed complete culture medium for 16 h at 37°C, in which time the parasites developed to late trophozoites. 10,000 Freshly prepared late trophozoites were injected i.v. into the donor monkey. Daily finger pricks were taken from day 3 onwards. Parasitaemia was monitored until it reached 1% (day 8), and 10 mL of blood was drawn. The donor monkey was subsequently cured with chloroquine (10 mg/kg i.m., Resochin®, Bayer, Leverkusen, Germany) and pyrimethamine (1 mg/kg per os, Sigma, Zwijndrecht, The Netherlands) for three consecutive days. Cells were extensively washed with RPMI 1640, and parasite infected cells were adjusted to 1×10^4^ mL^−1^. All monkeys received 1 mL of this parasite suspension containing 10,000 trophozoites i.v. in the vena sapphena, followed by 5 mL of PBS to ensure the whole inoculum was delivered. Finger prick blood samples were taken daily starting from 3 days post infection and continued for three weeks for the first challenge and five weeks for the second and third challenge. Thin blood smears were prepared every day at approximately 15:00h when the majority of parasites were at ring stage. Parasitaemias were determined by counting at least 4,000 erythrocytes in Giemsa-stained thin blood smears. Slides with very low counts of infected red blood cells (iRBC) were arbitrarily assigned a parasitaemia of 0.005%. Animals were cured if the levels of parasitaemia reached ≥2% or at parasitaemias between 1 and 2% if high numbers of schizonts were observed. Treatment was by i.m. injection of chloroquine and pyrimethamine as described. At the end of each challenge phase (21 days for the primary or 35 or 34 days for the second and third challenges, respectively), all animals not yet cured were then treated with chloroquine and pyrimethamine as described.

### Antibody ELISA

96-well microtitre ELISA plates were coated with 50 ng protein per well and incubated overnight at 4°C. Plates were washed and blocked. Serum samples were diluted in serial two fold dilutions, starting at 1/5,000 and were in duplicate added to antigen-coated wells and incubated for 1 h at 37°C. A duplicate control dilution series of a standard rabbit or rhesus anti PkAMA1 standard serum pool was prepared from immune serum and included on every plate. After extensive washing, the plates were incubated with secondary antibodies goat anti-rabbit or anti-human immunoglobulin G conjugated to alkaline phosphatase (Pierce, Rockford, IL). Bound antibodies were visualized by adding the substrate solution pNPP (*para* nitro phenyl phosphate, Sigma, Zwijndrecht, The Netherlands) and incubated for 30 minutes at room temperature. Absorbance at 405 nm was measured with a Bio-Rad Microplate Reader 3550 (BioRad, Veenendaal, The Netherlands). A standard curve was constructed by four-parameter curve fitting for every plate and titres of unknowns were calculated from the four-parameter fit. Antibody titres were expressed as arbitrary units (AU), where 1 AU corresponds to an amount of IgG theoretically yielding an OD of 1 over background. Thus the titre in AU indicates the dilution at which an OD of 1 over background is achieved.

### Growth inhibition assay

The *in vitro* anti-parasite activity of total IgG was determined by *P. knowlesi* growth inhibition assays (GIA). Total IgG from serum or plasma was purified on Protein A columns using binding and elution buffers supplied by the manufacturer (Pierce, Rockford, IL), exchanged into RPMI 1640 and concentrated to either 12 mg mL^−1^ for rabbit IgG or 20 mg mL^−1^ for monkey IgG using ultra centrifugal devices (Millipore, Etten-Leur, The Netherlands), and subsequently filter sterilised through a 0.22-µm filter (Millipore, Etten-Leur, The Netherlands). IgG antibodies were aliquoted and frozen at −20°C until use. An alanine-synchronised late stage (schizont) *P. knowlesi* (H-strain) culture was adjusted to 1% parasitaemia and 4% haematocrit in 40% normal human serum and 30 µg mL^−1^ gentamycin. Purified IgG was added in a volume of 30 µl in a 96-well flat-bottomed tissue culture plate (Greiner, Alphen a/d Rijn, The Netherlands), to which 30 µL of the parasite culture was added. Samples were tested in triplicate at four IgG concentrations; viz. 6, 3, 1.5 and 0.75 mg mL^−1^ for rabbit or 10, 5, 2.5 and 1.25 mg mL^−1^ for rhesus IgG. The highest IgG concentration (10 mg mL^−1^) used for rhesus GIA corresponds to the total IgG concentration in undiluted rhesus serum, thus GIA activity at 10 mg mL^−1^ can be interpreted as an estimate of GIA activity in undiluted rhesus serum. The inhibitory mAb R3/1C2 (1.5 mg mL^−1^) and EDTA (4 mM) were also included in every test plate as positive controls. The final concentration of the culture was 1% parasitaemia at 2% haematocrit and 20% normal human serum. Plates were incubated at 37°C under a 5% O_2_, 5% CO_2_, 90% N_2_ moist atmosphere for one cycle (26–30 h). Every assay was routinely performed in triplicate. Parasite specific lactate dehydrogenase (pLDH) activity, a measure for the total number of parasites, was measured using standard protocols [32].

Inhibition was calculated as 100×[1−((OD_650_ of infected RBCs with tested IgG−OD_650_ of uninfected RBCs)/(OD_650_ of infected RBCs with pre-immune IgG−OD_650_ uninfected RBCs))]. GIA assays were performed in two separate batches; all assays up to and including the second challenge were done in one batch. GIA assays for the third challenge were done independently from the aforementioned series.

### Lymphocyte stimulation assays

PBMCs were isolated from heparinised peripheral blood by density gradient centrifugation using lymphocyte separation medium (Invitrogen, Leek, The Netherlands) at 2100 rpm for 15 minutes without brake at room temperature. Cells at the interphase were collected and washed twice with RPMI 1640 medium. The PBMCs were resuspended in 8% foetal calf serum (FCS) (ICN, The Netherlands) in RPMI 1640 medium and adjusted to 2×10^6^ cells mL^−1^. The cell suspension (100 µL) was added in triplicate to 96 well round bottom plates (2×10^5^ cells well^−1^). One-hundred µL culture medium containing AMA1 antigen was added to a final AMA1 concentration of 10 µg mL^−1^. Concanavalin A was added as positive control at a final concentration of 5 µg mL^−1^. Cells were incubated at 37°C in 5% CO_2_ atmosphere for 3 days. Supernatant was harvested and stored at −20°C until further use.

### Cytokine measurement by ELISA

Culture supernatants were thawed at room temperature and assayed for IFN-γ and IL-13 using macaque IFN-γ and IL-13 ELISA kits (U-Cytech Biosciences, Utrecht, The Netherlands) according to the manufacturer's instructions. Recombinant IFN-γ or IL-13 were used as standards. Concentrations of unknowns were estimated using a four-parameter fit on the standard curve. Data are presented as background corrected values, i.e. background (medium only) is subtracted from the stimulated values.

### Relation between GIA and *in vivo* parasite growth

*In vivo* growth was estimated for all animals. The initial parasitaemia was calculated by dividing the inoculum (10,000) by the total numbers of red blood cells (estimated by weight and average RBC) and multiplied by four (assuming four infectious merozoites arise from one trophozoite). The parasitaemia observed at patency was then divided by the initial parasitaemia, yielding the multiplication factor between infection and day of patency plus one. The average per day *in vivo* growth rate was then calculated by taking the day of patency^th^ root of the multiplication factor. In the event animals became patent, without increasing parasitaemia in the days following patency, the last occurrence of patency observed was used for calculations. This yields a conservative estimate, as it could be argued that these animals did not require treatment and thus were protected and could be analysed similar to animals that did not become patent at all. Animals that did not become patent were arbitrarily assigned the last day of observation. Average per day *in vivo* growth rates in controls for the three separate challenges were calculated, excluding the non-responder animal for the first two challenges, and were used for subsequent *in vivo* growth inhibition (IVGI) calculations. IVGI was calculated as follows: 100 × (1−(growth in PkAMA1 animal/average growth in PfAMA1 group)).

### Statistical analysis

All statistical analyses were performed with the R language and environment for statistical computing (R Foundation for Statistical Computing, Vienna, Austria. ISBN 3-900051-07-0, URL http://www.R-project.org). Evaluation of statistical significance for IgG levels was performed on log-transformed titres and IgG titres are presented as geometric means with 95% confidence intervals (95% CI). Differences in GIA titres were analysed by t-test and GIA titres are presented as arithmetic means with 95% CI (the percent sign used in conjunction with GIA titre differences throughout the paper should be interpreted as percent points). IFN-γ and IL-13 levels are presented as medians with P_25_ and P_75_ percentiles, between group comparisons were done using the non-parametric Mann-Whitney test and paired data (between time points) were analysed using Wilcoxon signed rank test. GIA and log-transformed IgG between time-point comparisons were done using paired t-tests. Survival data (time to patency or time to drug cure) were evaluated using log-rank tests.

The relation between IgG and the GIA titres was investigated by non linear least squares regression using the following formula: GIA = 100 * 1/[1 + Exp((Ln IC_50_−Ln IgG) * Slope)], where IgG is the antibody titre (PkAMA1 specific IgG in AU mL^−1^ or total IgG in mg mL^−1^) and IC_50_ represents the IgG concentration yielding 50% inhibition. Slope is a parameter indicating the steepness of the curve, with steeper curves at higher values. As Slope is rather difficult to interpret it is expressed as the fold-increase in IgG required to raise GIA levels from 10 to 90%. (It can be shown algebraically that this is: e^[ln(9) – ln(1/9)]/Slope^, which is approximately e^(4.394/Slope)^ and for an increase from 10 to 50% or 50 to 90% this is approximately e^(2.197/Slope)^). IC_50_ and Slope values were estimated from GIA data for each animal at 4 different total IgG concentrations (viz. 1.25, 2.5, 5 and 10 mg mL^−1^) using a non-linear mixed effects model employing the GIA parameterisation described above. This method reduces 4 GIA data points at different total IgG concentrations to two parameters (viz. IC_50_ and slope) and thus makes full use of the GIA titration data. The IC_50_ value represents the amount of total IgG required to obtain 50% growth inhibition and can thus be considered as a measure of GIA activity in a serum, with lower IC_50_ values representing more potent GIA activity. The results of the fitting procedure are depicted in Figure S1, and IC_50_ values are indicated.

## Results

### Production, purification and quality control of PkAMA1

**Figure 2:**
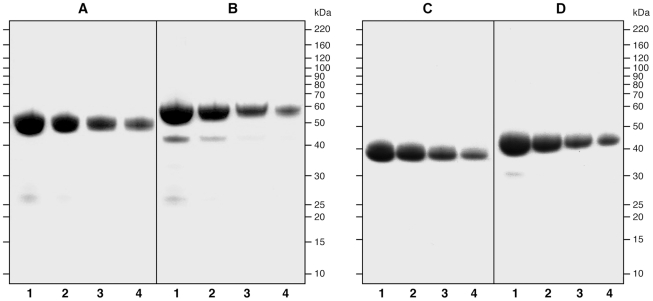
SDS-PAGE gel of vaccine antigens. SDS-PAGE gel of *Pichia pastoris* produced PkAMA1 DI-II-III, A non-reduced and B reduced, PkAMA1 DI-II, C non-reduced and D reduced. Lane 1, 10 µg; lane 2, 5 µg; lane 3, 2.5 µg; lane 4, 1.25 µg.

The *Pichia pastoris* expression system was used to produce AMA1 from *P. knowlesi* H strain (amino acids 43–487 and aa 43–387), with potential N-glycosylation sites removed. Several runs in a 7-Liter fermentor were performed and subsequently the protein was purified using Ni-IMAC, as the protein contains a hexa-histidine tag. Gel filtration was used in the second step, essentially for buffer exchange and for the removal of excess of dimers. Yields were approximately 2 and 40 mg mL^−1^ for PkAMA1 D I-II-III and PkAMA1 D I-II, respectively.

table-2-captionThe apparent molecular masses of the two constructs analyzed by 4–12% gradient SDS-PAGE were 50 kDa and 40 kDa for PkAMA1 DI-II-III and PkAMA1 DI-II respectively, in accordance with theoretical mass calculated from their respective amino acid sequences. Figure 2 shows that both products are near to apparent homogeneity. Reduction of PkAMA1 D I-II-III protein samples resulted in the appearance of two additional low molecular weight bands (Figure 2). The apparent size of the two smaller products is in accordance with the location of a proteolytic cleavage site in DII, observed in PfAMA1 FVO and 3D7 [25], [32]. Densitometric analysis of SDS-PAGE on the non-reduced samples revealed that 96% of the protein was in the main band for PkAMA1 DI-II-III and 99% for PkAMA1 DI-II. Densitometry on the reduced samples showed that less than 10% and 3% of the protein is internally cleaved, for PkAMA1 DI-II-III and PkAMA1 DI-II, respectively. Both proteins react with anti hexa-histidine antibodies, anti myc-antibodies and with the PkAMA1 specific antibody R3/1C2 (data not shown), as expected.

As it has been shown that immunisation with reduced and alkylated AMA1 results in low functional antibody titres [33], we designed an assay to assess the conformational intactness of the protein, by a quantitative binding assay to immobilized R3/1C2, essentially as described before [25]. R3/1C2 recognizes a reduction-sensitive epitope [4]. Eighty-eight (88%) percent of PkAMA1 (DI-II-III) was bound to immobilized R3/1C2.

### Comparative immunogenicity studies and growth inhibition in rabbits using the novel adjuvant CoVaccine HT™

The protein products made were first tested for immunogenicity in rabbits. These experiments were, given the low yield of the full length PkAMA1, also used to select the antigen of choice for the rhesus study, as unpublished studies by ourselves and those of Lalitha and co-workers [34] suggest that PfAMA1 domain I and II is nearly as good as PfAMA1 domain I, II and III at inducing growth inhibiting antibodies. To this end, two groups of five rabbits were immunised with 50 µg of either PkAMA1 DI-II-III or PkAMA1 DI-II adjuvanted with CoVaccine HT™. Immunisations were given at days 0, 28 and 56, and a final bleed was taken at day 70. Unfortunately one rabbit in the PkAMA1 DI-II group died during the immunisation phase from causes not related to the vaccination. Titres to full length PkAMA1 (Domains I, II & III) were similar in both groups with 370.6 (95% CI 205.4 to 670.0) and 315.4 (95% CI 169.0 to 588.8) kAU mL^−1^ for PkAMA1 (DI- II-III) versus (DI-II) respectively (t-test, p = 0.595). The same observation was made for PkAMA1 domains I & II. Geometric mean titres were 205.9 (95% CI 108.8 to 389.7) and 256.9 (95% CI 140.2 to 470.8) kAU mL^−1^ for PkAMA1 DI-II-III versus Pk DI-II, respectively (t-test, p = 0.483). At an IgG concentration of 6 mg mL^−1^, GIA titres were, however, significantly higher in the PkAMA1 (DI-II-III) group at 64.5% (52.8 to 76.3) versus 43.5% (24.0 to 62.9) in the PkAMA1 (D I & II) group (t-test, p = 0.032). This trend was consistent for 3, 1.5 and 0.75 mg mL^−1^ IgG (t-test, p = 0.019, 0.033 and 0.053, respectively). Based on these results, it was decided to use PkAMA1 DI-II-III for the rhesus vaccinations.

### Safety and local reactions in rhesus macaques

All 12 rhesus monkeys in the study were carefully observed in their behaviour (general, vividness, appetite) and monitored for their general health (weight, stool) during the full course of the study. At day 1, 7 and 14 following the vaccinations at days 0, 28 and 56, blood samples were taken and analysed for haematological and clinical chemistry parameters. Both vaccine formulations were well tolerated. No vaccine related serious adverse events (i.e. an event which hypothetically might have caused death if it were more severe) were observed. For all but two animals, body weights remained unchanged during the observation period. One animal in the PkAMA1 group had a decreased appetite between days 8 and 15. No oedema, erythema or hypersensitivity reactions were observed at the injection sites. Following vaccination three animals in PkAMA1 group and one animal in PfAMA1 had a slight swelling in the draining inguinal lymph node that resolved within two weeks. Three animals in the PfAMA1 group developed a transient muscle induration at the site of injection following vaccination. After each immunisation, one animal in the PfAMA1 group experienced muscle induration at the point of injection with a maximum size of 2.5 cm. These indurations resolved within two to four weeks and did not influence the animals' behaviour.

In general, none of the animals showed persistent deviations from the reference values during the course of the study. Acute effects of sedation and sampling were evident, and are generally observed during vaccination studies. Elevations following vaccination were observed in white blood cell count, basophil count and monocytes. These returned to baseline values within 14 days after each vaccination. Neutrophil levels increased to above normal values with a concomitant drop in free serum iron. Both resolved within 14 days after immunisation.

### Blood-stage parasite challenge

**Figure 3:**
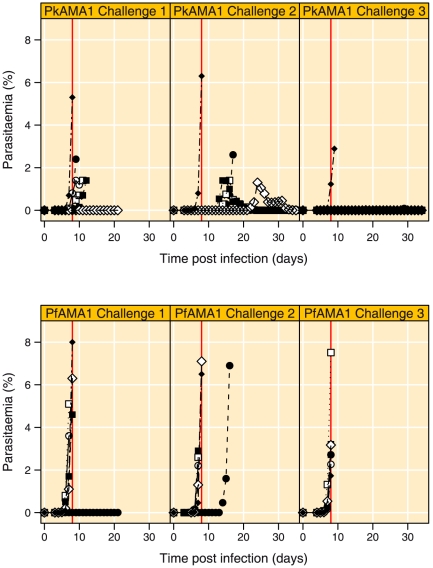
Parasitaemia development following i.v. challenge with *P. knowlesi* blood stage parasites. Parasitaemia development versus time during the 3 challenges. Top panels are PkAMA1-immunised experimental animals and bottom panels PfAMA1-immunised control animals. Day 8 post challenge is indicated by a vertical red line. The symbols within each treatment group refer to the same animals throughout all figures.

At day 70, all animals were blood stage challenged by *in vivo* passage of 10,000 *P. knowlesi* H strain late trophozoites. The left hand panels in figure 3 show the parasitaemia in the blood of individual animals during the course of infection, both in the immunised (top panel) and control (bottom panel) group. Table 2 shows the parasitological and immunological data at the day of challenge. Five animals from the control group had to be cured within 8 days, the sixth animal, however (R01041 [•]) did not become patent during the challenge phase (Figure 3, Table 2). One monkey from the PkAMA1 group, (Ri9980203 [⋄]) was patent at days 10 and 14 post infection, but was obviously able to control the parasitaemia (Table 2). The other 5 animals had to be cured, but there was an average delay of about 2 days in the day of onset of the parasitaemia between the PkAMA1 group and the PfAMA1 control group, albeit not statistically significant (log-rank test, p = 0.453). The time to drug cure was also delayed for 4 out of 6 animals, but again not statistically significant (log-rank test, p = 0.28). When these analyses were repeated excluding the control monkey that did not become patent (R01041 [•]), both time to patency and time to drug cure were significantly longer in the PkAMA1 vaccinated group (log-rank test, p = 0.006 and 0.002, respectively).

**Table 2:** Parasitological and immunological data at the time of challenges.

Monkey	Symbol	Protected	DoP	DoT	*in vivo* Growth	IVGI	Pf IgG	Pk IgG	GIA10	IC50	IC10–90
PkAMA1-immunised (verum) animals											
First Challenge											
R00066	○	No	6	10	4.34	35.2	7.83	50.00	62.75	6.27	10.67
R02013	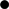	No	7	9	3.84	42.7	32.89	93.89	75.75	4.90	8.29
R97025	□	No	7	11	5.06	24.4	14.27	38.53	76.41	5.47	9.21
Ri9902011	▪	No	9	12	2.85	57.4	51.52	142.53	79.42	4.76	8.07
Ri9980203	⋄	Yes	10/14	21	1.84	72.5	35.01	77.57	108.05	3.33	6.12
Ri9902033	⧫	No	6	8	6.21	7.3	21.56	61.56	67.90	7.66	13.74
Second Challenge											
R00066	○	Yes	34	35	1.36	74.1	39.42	162.81	98.71	1.66	11.19
R02013	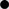	No	9/13	17	2.07	60.6	51.32	176.47	93.51	2.64	10.36
R97025	□	Yes	7/12	35	2.02	61.6	47.09	168.62	89.36	2.66	10.35
Ri9902011	▪	Yes	10	35	2.32	55.7	49.35	126.28	96.46	3.01	10.12
Ri9980203	⋄	Yes	18	35	1.63	69.0	50.04	129.95	104.64	1.67	11.20
Ri9902033	⧫	No	5	8	5.61	−7.0	34.43	50.79	37.11	12.10	8.26
Third Challenge											
R00066	○	Yes	15/19/24	34	1.51	72.6	45.7	135.7	85.5	0.94	119.8
R02013	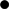	Yes	16/25/29	34	1.44	73.8	44.8	168.3	89.5	3.40	15.4
R97025	□	Yes	18/21/22	34	1.48	73.1	60.1	230.9	82.1	2.71	19.2
Ri9902011	▪	Yes	14	34	1.93	65.0	129.5	365.9	83.7	1.72	34.2
Ri9980203	⋄	Yes	6/18	34	1.65	70.2	52.9	203.9	83.0	0.54	1166.2
Ri9902033	⧫	No	7	9	5.35	3.0	33.8	56.9	45.3	11.32	7.0
PfAMA1-immunised (control) animals											
First Challenge											
R00080	○	No	5	7	6.89	−2.9	132.9	22.2	7.4	nc	nc
R01041	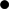	No	21	21	1.46	78.2	116.4	38.8	3.9	nc	nc
R01077	□	No	5	7	7.77	−16.1	163.1	50.7	−2.4	nc	nc
Ri10177	▪	No	5	8	7.35	−9.8	293.7	27.0	5.4	nc	nc
Ri397	⋄	No	5	8	6.13	8.4	266.6	170.2	1.2	nc	nc
Ri9806051	⧫	No	5	8	5.33	20.4	164.0	44.2	−4.2	nc	nc
Second Challenge											
R00080	○	No	5	7	4.99	4.9	206.6	18.3	10.2	nc	nc
R01041	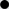	No	7/12	16	1.94	63.0	141.1	17.5	−16.9	nc	nc
R01077	□	No	5	7	5.50	−4.9	160.7	31.0	−17.1	nc	nc
Ri10177	▪	No	5	7	5.69	−8.4	141.0	32.0	−13.7	nc	nc
Ri397	⋄	No	5	8	4.79	8.7	159.0	27.4	−4.5	nc	nc
Ri9806051	⧫	No	5	8	5.26	−0.3	144.9	40.8	21.4	nc	nc
Third Challenge											
R00080	○	No	7	8	5.34	3.1	394.5	71.4	−8.4	nc	nc
R01041	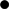	No	6	8	4.41	20.0	161.8	9.8	6.6	nc	nc
R01077	□	No	5	8	7.95	−44.3	323.3	23.9	−4.9	nc	nc
Ri10177	▪	No	6	8	6.37	−15.4	188.0	32.3	11.6	nc	nc
Ri397	⋄	No	6	8	4.51	18.1	212.5	11.9	−11.3	nc	nc
Ri9806051	⧫	No	6	8	4.49	18.5	174.2	26.8	9.2	nc	nc

DoP = day of patency multiple patency events are separated by a slash, DoT = day of treatment, *in vivo* Growth = estimated *in vivo* multiplication per cycle, IVGI = *in vivo* growth inhibition calculated as outlined in materials and methods, Pf IgG and Pk IgG = the Pf and Pk IgG titre, respectively, at the time of parasite challenge, GIA10 = the observed GIA titre at 10 mg mL^−1^ total IgG at the time of parasite challenge, IC_50_ = the amount of total IgG (mg mL^−1^) required for 50% inhibition at the time of parasite challenge, IC10–90 = the fold difference between the total IgG concentrations required for 10 and 90% GIA titres at the time of parasite challenge. Slope = Parameter that indicates the steepness of the sigmoid GIA versus IgG curve. nc = Not Calculated.

### Immunological analysis of the first phase of the study

**Figure 4:**
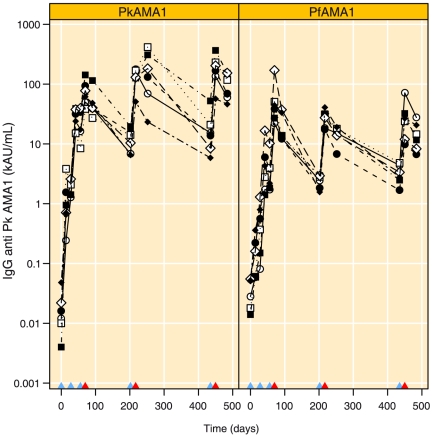
IgG antibody levels to the PkAMA1 vaccine antigen. Blue triangles on the x-axis indicate immunisations and red triangles indicate time of parasite challenge. PkAMA1 = PkAMA1-immunised experimental animals, PfAMA1 = PfAMA1-immunised control animals.

Figure 4 shows that all animals had low anti-PkAMA1-IgG levels before vaccination (14 95% CI 6 to 34 and 32 95% CI 17 to 61 AU mL^−1^, for PkAMA1 and PfAMA1 groups respectively). Following vaccination, animals in the PkAMA1 group had detectable levels of antibodies to PkAMA1 two weeks after the first immunisation (939, 95% CI 358 to 2460 AU mL^−1^, paired t-test with day 0, p = 0.0006). Anti PkAMA1-IgG also increased in the PfAMA1 group, but at a much lower rate (118, 95% CI 51 to 271, paired t-test with day 0, p = 0.0005). The second immunisation at day 28 boosted responses at day 56 to high levels in the PkAMA1 group (22.3, 95% CI 11.7 to 42.6 kAU mL^−1^, paired t-test with day 28, p = 0.0002). PkAMA1-IgG levels in the PfAMA1 group were also boosted significantly after the second immunisation (3.2, 95% CI 1.6 to 6.5 kAU mL^−1^, paired t-test with day 28, p = 0.0006). At day 56 anti-PkAMA1-IgG levels were significantly higher in the PkAMA1 group (7.0 fold, 95% CI 3.0 to 16.0 fold, t-test p- value = 0.0004). The third vaccination at day 56 boosted anti PkAMA1-IgG levels at day 70 even further; animals in the PkAMA1 group had 70.5 kAU mL^−1^ (95% CI 43.2 to 115.1, paired t-test with day 56, p = 0.0002) and animals in the PfAMA1 had 45.5 kAU mL^−1^ (95% CI 21.5 to 96.5, paired t-test with day 56, p<0.0001). This was in part due to one animal (Ri397 [⋄]) in the PfAMA1 group that had an unexpectedly high anti PkAMA1 titre (164.0 kAU mL^−1^) at day 70 and this value was confirmed in repeat ELISA's. In parallel, the IgG titre to the PfAMA1 vaccine antigen was also high in this animal (266.6 kAU mL^−1^). Day 70 IgG levels to PkAMA1 were not significantly higher in the PkAMA1 group [70.5 (95% CI 43.2 to 115.1) versus 45.5 (95% CI 21.5 to 96.5) kAU mL^−1^ (p = 0.24)] as compared to the PfAMA1 group. IgG levels to PkAMA1 decreased during the challenge phase in both groups (PkAMA1 group: paired t-test with day 70, p = 0.0122 to 45.9 kAU mL^−1^ 95% CI 27.5 to 76.5 and PfAMA1 group: paired t-test with day 70, p = 0.0052 to 18.1 kAU mL^−1^ 95% CI 10.5 to 31.3).

**Figure 5:**
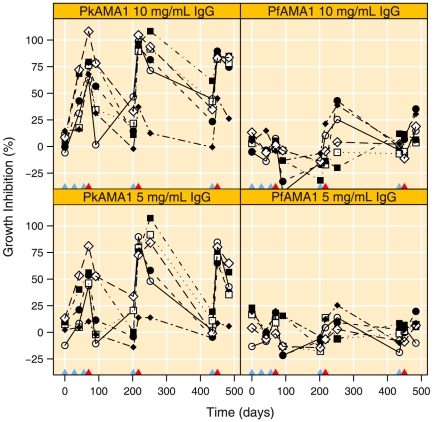
Parasite Growth Inhibition by purified IgG antibodies. GIA titres with *P. knowlesi* H-strain at 10 and 5 mg mL^−1^ IgG for PkAMA1 immunised experimental group and PfAMA1 immunised controls. Blue triangles on the x-axis indicate immunisation time points, red triangles indicate time of parasite challenges.

Figure 5 shows the GIA titres in the two treatment groups. The first two immunisations at days 0 and 28 induced clearly detectable GIA activity in the PkAMA1 vaccinated animals and GIA levels had reached 41.3% at day 42 (95% CI 15.6 to 67.0, paired t-test with day 0, p = 0.0218) between days 0 and 42 (figure 5). By contrast GIA levels in the PfAMA1 group did not change significantly (−2.7%, 95% CI −2.9 to 6.6% at day 42, paired t-test with day 0, p = 0.226) (figure 5). The third immunisation increased GIA levels even further in the PkAMA1 group to 78.4% at day 70 (95% CI 61.8 to 94.9%, paired t-test with day 42, p = 0.003), while GIA levels remained unchanged in the PfAMA1 group 1.9% (, 95% CI −12.6 to 7.2%, paired t-test with day 42, p = 0.437). At the time of challenge (day 70) GIA levels were significantly higher in the PkAMA1 group as compared to the PfAMA1 control group (t-test, p<0.0001) (figure 5). GIA levels in the PkAMA1 group decreased significantly during the challenge phase to 46.7% (95% CI 15.2 to 78.3%, paired t-test with day 70, p = 0.0128) and a small non-significant decrease was observed for animals in the PfAMA1 group; −18.2% at day 91 (−95% CI −35.1 to −1.4%, paired t-test with day 70, p = 0.053).

**Figure 6:**
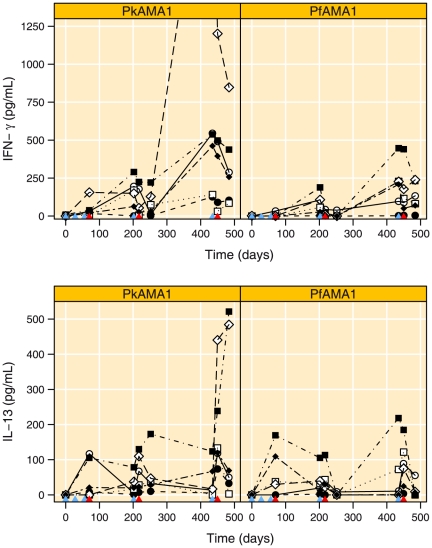
IFN-γ and IL-13 production following *in vitro* stimulation with PkAMA1. Blue triangles on the x-axis indicate immunisation time points, red triangles indicate time of parasite challenges. PfAMA1 = PfAMA1-immunised control animals, PkAMA1 = PkAMA1-immunised experimental animals. The point not depicted for PkAMA1 [⋄] is at 2757 pg mL^−1^ IFN-γ.

Antigen-specific lymphocyte stimulation assays showed that IFN-γ levels were generally low with a median values of 25.4 (Quartile range: 17.7 to 36.3 pg mL^−1^) and 1 (Quartile range 1 to 1 pg mL^−1^) for the PkAMA1 and PfAMA1 groups, respectively. The difference in IFN-γ levels was statistically significant (p = 0.025, Mann-Whitney test). The protected monkey in the PkAMA1 group produced the highest IFN-γ level upon stimulation with PkAMA1, while all other samples were low to negative in this assay (Figure 6). IL-13 levels were low; the median IL-13 values were 14.5 (Quartile range 2.8 to 84.2 pg mL^−1^) and 33.8 (Quartile range 8.2 to 91.5) for the PkAMA1 and PfAMA1 groups, respectively (p = 0.68, Mann-Whitney test). The IL-13 level of the protected monkey was not elevated; in the PkAMA1 group only two monkeys (R00066 [○] and Ri9902011 [▪]) had elevated levels of IL-13 following stimulation with PkAMA1 (figure 6). The animal in the control group that did not become patent did not produce detectable IFN-γ nor IL-13 upon stimulation with PkAMA1. Four out of two control monkeys (Ri10177 [▪], Ri9806051 [⧫], Ri397 [⋄] and R01077 [□]) in the control group produced some IL-13, but no detectable IFN-γ.

### *Plasmodium knowlesi* blood-stage challenge after booster vaccination

The results described above obtained after the primary challenge prompted us to extend the study with an additional boost and second challenge, similar to Deans and co-workers [6]. Vaccine and control animals received one additional booster immunisation of 50 µg PkAMA1 or PfAMA1, respectively, 203 days following the first vaccination. At day 217 the animals were re-challenged with 10,000 *P. knowlesi* H strain parasites. The parasitaemias for the second challenge are depicted in the top middle and bottom middle panels of figure 3 and parasitological and immunological data are provided in table 2.

Five out of 6 control monkeys were chloroquine-treated before or at day 8 post-challenge. The sixth animal (R01041 [•]), the same monkey that did not become parasitaemic after the first challenge, became patent on days 7 and 12; it had a delay of the onset of parasitaemia, but required treatment at day 16 post-challenge (Figure 3).

Four rhesus monkeys in the PkAMA1 group were able to control the parasitaemia (Figure 3). In these four animals the parasitaemia onset varied between day 7 (R97025 [□]) and day 34 (R00066 [○]). One rhesus monkey (Ri9902033 [⧫]) in the PkAMA1 group became patent 5 days post challenge and was treated 8 days post-challenge, the sixth monkey (R02013 [•]) became patent at day 9 and was treated on day 17 (Figure 3, Table 2). Time to patency and time to drug cure were significantly delayed in the PkAMA1 group (p = 0.009 and 0.0029, respectively).

### Immunological analysis of the second challenge

On the day of re-boost, anti-PkAMA1-IgG antibodies had decreased significantly in both groups compared to the levels after challenge (day 91) (PkAMA1 group: 11.2 kAU mL^−1^, 95% CI 7.0 to 18.0, paired t-test with day 91, p = 0.0013 and PfAMA1 group:2.4 kAU mL^−1^, 95% CI 1.8 to 3.1, paired t-test with day 91, p = 0.0052) (Figure 4). Boosting was effective and at the time of the second challenge (day 217) IgG levels had risen to 126.2 kAU mL^−1^ (95% CI 77.3 to 206.0, paired t-test with day 202, p<0.0001) and GIA levels had increased to 86.6% in the PkAMA1 group (95% CI 60.6 to 112.7%, paired t-test with day 202, p = 0.0002) (Figures 4 and 5). Anti-PkAMA1-IgG levels in the PfAMA1 group showed an increase to 26.6 kAU mL^−1^ (95% CI 18.8 to 37.8, paired t-test with day 202, p<0.0001). GIA levels increased only slightly in the PfAMA1 group to −3.4% (95% CI −20.2 to 13.4%, paired t-test with day 202, p = 0.0530). At the time of challenge IgG and GIA levels were significantly higher in the PkAMA1 group (IgG: 4.7-fold, 95% CI 2.8 to 8.1-fold, t-test, p<0.0001 and GIA: 90.1% higher, 95% CI 62.6 to 117.5%, p<0.0001).

IgG antibody levels and GIA titres in the PkAMA1 group were slightly higher compared to the values obtained on day 70 (IgG levels: 1.79-fold 95% CI 0.884 to 3.62-fold, paired t-test, p = 0.0875 and GIA 8.2% higher, 95% CI −15.8 to 32.3, paired t-test, p = 0.42). On day 252 at the end of the second challenge, anti PkAMA1-IgG levels were unchanged in the PkAMA1 group (1.03-fold, 95% CI 0.45 to 2.4, paired t-test, p = 0.92) and significantly decreased in the PfAMA1 group (0.52-fold, 95% CI 0.40 to 0.69, paired t-test, p = 0.00172).

For the PkAMA1 immunised group, GIAs done on IgG obtained five weeks after the second challenge did not decrease as markedly as after the first challenge (10% decrease, 95% CI −5.8 to 26.0%, paired t-test, p = 0.163) (figure 5). Conversely, GIA levels increased during the challenge in the PfAMA1 group (17.8% increase, 95% CI −5.6 to 41.2%, paired t-test, p = 0.1086). The four protected animals in the PkAMA1 group had low GIA IC_50_ values at the time of challenge. The animal (R02013 [•]) with a low IC_50_ of at 2.6 mg mL^−1^ at the time of challenge was drug cured on day 17, and the animal (Ri9902033 [⧫]) with the highest IC_50_ was drug cured 8 days after challenge (Table 2).

IFN-γ levels did not change significantly between days 70 and 202 in the PkAMA1 group with a median value of 159.9 (Quartile range: 84.5 to 189.0 pg mL^−1^) (Wilcoxon signed rank test, p = 0.16). In the PfAMA1 group, however, IFN-γ levels increased significantly between days 70 and 202 to 81.4 (Quartile range: 34.4 to 107.2 pg mL^−1^. Day 202 IFN-γ levels were not statistically different between the two groups (Mann-Whitney U test, p = 0.13) (Figure 6). IFN-γ levels decreased in both groups following booster vaccination (day 217) (Wilcoxon signed rank test on pooled data for PkAMA1 and PfAMA1, p = 0. 0015), with median values of 40.1 (Quartile range: 14.3 to 147.1 pg mL^−1^) and 8.4 (Quartile range: 6.3 to 18.0 pg mL^−1^), for PkAMA1 and PfAMA1 groups, respectively. IL-13 levels did not change appreciably between days 70 and 202 in either group (Wilcoxon signed rank test, p = 0.47) (Figure 6). Booster vaccination resulted in an increase in IL-13 levels in both groups (Wilcoxon signed rank test on pooled data for PkAMA1 and PfAMA1, p = 0.012). The two monkeys in the PkAMA1 group that required drug treatment had low IFN-γ levels at the day of challenge, whereas three out of the four protected monkeys had clearly detectable IFN-γ levels at the time of challenge. The monkey in the control group (R01041 [•]) with a delay in parasitaemia did not have detectable levels of IFN-γ nor IL-13 (Figure 6).

### Protection after re-boost and heterologous blood-stage challenge with Pk Nuri strain

To investigate whether the protection observed upon the second challenge was AMA1-mediated, monkeys were boosted with Pk or PfAMA1 and challenged with the Pk Nuri strain. The Pk Nuri AMA1 differs by only one amino acid from the H-strain, whilst other antigens expressed on the surface of infected erythrocytes that may contribute to protective responses (e.g. Schizont-Infected Cell Agglutination, SICA-Var) are expected to be different in a heterologous strain [11]. The heterologous challenge thus applies to parasite antigens other than AMA1, a situation considerably different from *P. falciparum* where the chance of finding two very closely related AMA1 molecules in different isolates is very small. The parasitaemias for the third challenge are depicted in the top right and bottom right panels of figure 3 and parasitological and immunological data are shown in table 2. All animals in the control group required treatment by day 8, while five out of six PkAMA1 vaccinated rhesus monkeys were able to control the parasitaemia, albeit not sterilely (Figure 3, Table 2). Low numbers of unhealthy appearing parasites were occasionally observed in these five animals (R00066 [○] days 15, 19 and 24, R02013 [•] days 16, 25 and 29, R97025 [□] days 18, 21 and 22, R9902011 [▪] day14 and R9980203 [⋄] days 6 and 18) (Table 2). Time to patency and time to drug cure were significantly longer in the PkAMA1 group (log-rank test, p = 0.009 and 0.0009 for patency and cure, respectively). One monkey in the PkAMA1 group (R02033 [⧫]) could not control parasitaemia and required drug cure by day 9. This was the same monkey that did not show any signs of protection in the two previous challenges.

### Immunological analysis of the third challenge

On the day of re-boost, anti-PkAMA1-IgG antibodies had decreased significantly to 15.0 kAU mL^−1^ (95% CI 6.8 to 33.4, paired t-test with day 252, p = 0.0008) for the PkAMA1 group and to 3.1 kAU mL^−1^(95% CI 2.0 to 4.6, paired t-test with day 252, p<0.0001), for the PfAMA1 groups compared to the levels after the second challenge (Figure 4). Boosting was effective and at the time of the third challenge (day 450), PkAMA1-IgG antibody levels had increased to 167.9 kAU mL^−1^ in the PkAMA1 group and to 23.6 kAU mL^−1^ in the PfAMA1 group (95% CI 87.0 to 324.0, paired t-test with day 435, p<0.0001 and 11.1 to 50.1, paired t-test with day 435, p = 0.0004 for Pk and PfAMA1 groups, respectively). At the time of the third challenge, anti PkAMA1-IgG levels were 7.1-fold higher (95% CI 3.0 to 17.0, t-test, p = 0.0005) in the PkAMA1 group as compared to the PfAMA1 group. Anti PkAMA1-IgG levels decreased significantly during the challenge period in both groups; to 89.9 (95% CI 52.6 to 153.8, paired t-test with day 450, p<0.0001) in the PkAMA1 group and to 13.2 (95% CI 7.5 to 23.3, paired t-test with day 450, p = 0.007) for the PfAMA1 group.

GIA levels appeared lower at day 450 as compared to day 217, but as these were determined in two independent runs, direct comparisons between days 217 and 450 can, unfortunately, not be made. GIA levels in the PkAMA1 group increased significantly following booster vaccination to 78.2% (95% CI 61.0 to 95.3, paired t-test with day 435, p = 0.0007). By contrast, GIA levels in the PfAMA1 group remained unchanged at 0.5% (95% CI −9.8 to 10.8%, paired t-test with day 435, p = 0.88). During the challenge phase, there was a tendency for decreasing GIA levels in the PkAMA1 group to 70.3% (95% CI 47.2 to 95.3%, paired t-test with day 450, p = 0.073), whereas GIA levels in the PfAMA1 group increased significantly to 18.3% (95% CI 4.9 to 31.7%, paired t-test with day 450, p = 0.024).

IFN-γ levels increased significantly in both groups between the end of the second challenge (day 252) and the day of the second booster vaccination (day 435) (Wilcoxon signed rank test on pooled data for PkAMA1 and PfAMA1, p = 0.0005). Vaccination resulted in a significant decrease of IFN-γ production in both groups (Wilcoxon signed rank test on pooled data for PkAMA1 and PfAMA1, p = 0.001) (Figure 6). Levels of IFN-γ did not change significantly during the challenge phase (Wilcoxon signed rank test on pooled data for PkAMA1 and PfAMA1, p = 0.30). IL-13 levels did not change significantly between the end of the second challenge and the day of the second booster vaccination in both groups (Wilcoxon signed rank test on pooled data for PkAMA1 and PfAMA1, p = 0.92) (Figure 6). Booster vaccination, however, induced a significant increase in IL-13 levels in both groups (Wilcoxon signed rank test, p = 0.0024) (Figure 6). IL-13 levels appeared to decrease in both groups during the challenge phase, but this failed to achieve statistical significance (Wilcoxon signed rank test on pooled data for PkAMA1 and PfAMA1, p = 0.11) (Figure 6).

### Relation between IgG and GIA

**Figure 7:**
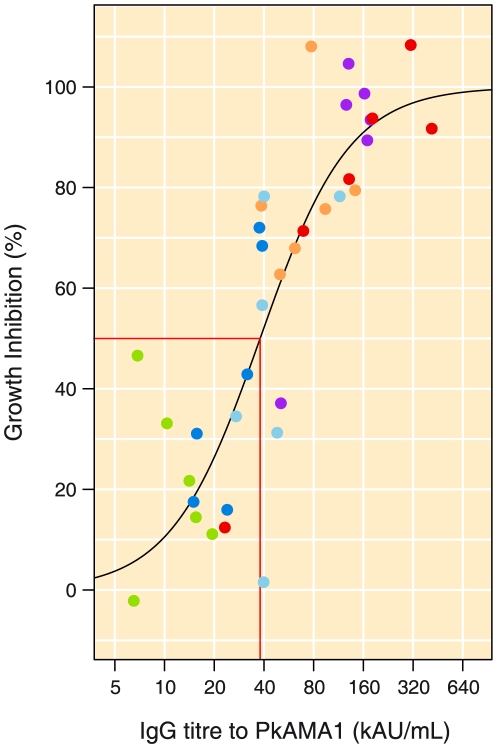
The relation between GIA and IgG titre. The IC_50_ value is indicated by red lines. The various colours represent the time points at which samples were taken: day 42 dark blue, day 70 orange, day 91 light blue, day 202 green, day 217 purple and day 252 red.

The relation between IgG and GIA was investigated by modeling a sigmoid curve using non-linear least squares regression. Day 0 samples from both groups were included and PkAMA1 vaccinated animals were used for time points thereafter. Analysis was restricted to GIA values from one GIA run at 10 mg mL^−1^ IgG, which corresponds to the amount of total IgG present in rhesus serum (days 42, 70, 91, 202, 217 and 252). Thus the amounts of GIA and PkAMA1-specific IgG both correspond to values obtained with undiluted serum or plasma. The relationship between GIA and PkAMA1-specific IgG is graphically presented in figure 7. The correlation coefficient was 0.908 (95% CI 0.841 to 0.948, p<0.0001). The IC_50_ value was estimated at 38 kAU mL^−1^ *P. knowlesi* AMA1-specific IgG (95% CI 31 to 46) and the fold difference in IgG between 10 and 90% GIA was estimated at 15.4 fold (95% CI 7.8 to 59.5) (Figure 7).

### Correlates of Protection: Relation between GIA and *in vivo* growth inhibition

**Figure 8:**
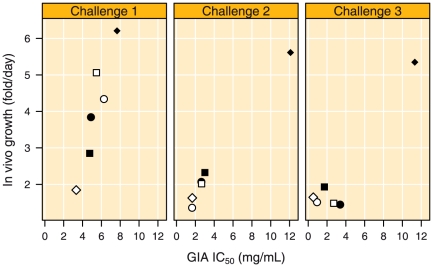
Relation between measured *in vitro* and estimated *in vivo* parasite growth. Relation between GIA IC_50_ values and estimated *in vivo* growth during the three intravenous parasite challenges.

During the first challenge estimated *In vivo* growth rate (Table 2 and Figure 8) was correlated with GIA IC_50_ values (Spearman's Rho = 0.94, p = 0.017). This correlation did not change when recalculated with the assumption that monkey Ri9980203 was patent on day 10 or considered protected. This correlation was also independent of the assumption that the inoculation of one late trophozoite yielded four infected red blood cells (iRBC). Changing number of iRBC arising from one inoculated late trophozoite, however, did change the estimated growth rates in the vaccinated and in the control animals that developed parasitaemia (8.8, 6.7 and 5.8 for 1, 4 or 8 iRBC arising from one inoculated trophozoite, respectively) and in the vaccinated animals as well.

The protected animal had an IC_50_ value of 3.3 mg mL^−1^ IgG (IVGI, 72.5%), and the three monkeys with a delayed onset of parasitaemia in the first challenge had IC_50_ values of 4.8 (day 9, IVGI, 57.4%), 4.9 (day 7, IVGI 42.7%) and 5.5 (day 7, IVGI 24.4%) mg mL^−1^ IgG, respectively. The two non-protected PkAMA1 animals, patent at day 6, had IC_50_ values of 6.3 (IVGI, 35.2%) and 7.7 (IVGI, 7.3%) mg mL^−1^ IgG, respectively (Table 2). The animal in the control group for which no parasites were detected did not have high levels of anti-PkAMA1 antibodies, nor detectable GIA activity.

The significant correlation between GIA IC_50_ values and *in vivo* growth was confirmed during the second challenge (Spearman's Rho = 0.94, p = 0.017) (Figure 8). When the correlation was recalculated using the first occurrence of patency for the estimation of *in vivo* growth rate, the correlation coefficient was 0.83 and not significant (p = 0.058). At the second challenge the four protected monkeys had IC_50_ values ranging between 1.7 and 3.0 mg mL^−1^ IgG; the monkey with a delay had an IC_50_ of 2.6 mg mL^−1^, whereas the non-protected animal had an IC_50_ value of 12.1 mg mL^−1^ IgG (Table 2).

At the third challenge the five protected animals all had IC_50_ values lower than 3.4 mg mL^−1^ IgG and the non-protected animal had an IC_50_ value of 11.1 mg mL^−1^ IgG (Table 2). The correlation between GIA IC_50_ and *in vivo* growth rate was, however, not observed at the third challenge (Spearman's Rho = 0.03, p = 1.0).

## Discussion

The rhesus *Plasmodium knowlesi* model enables the investigation of *in vivo* protection following (repeated) mosquito or blood-stage challenge with a longer observation window as compared to challenge studies in humans. The model also offers the opportunity to assess clinical safety in non-human primates. This safety evaluation includes clinical observations (behaviour, appetite injection site inspection etc.) as well as full haematology and clinical chemistry evaluations. Therefore the rhesus *Plasmodium knowlesi* model can be a valuable tool for the down-selection of human malaria vaccine candidates and formulations (provided a Pk orthologue is available).

The main findings of the work reported here are: i.) Vaccination with heterologously-expressed PkAMA1 formulated with a potent adjuvant protects rhesus monkeys from subsequent blood-stage challenge (i.e. animals are able to control parasitaemia). ii.) Protection appeared to be mainly antibody-mediated, although a contribution of cellular immunity cannot be excluded. iii.) High levels of growth inhibiting antibodies are required for protection. iv.) The degree of protection improves upon repeated infection and boosting. v.) Protection against blood-stage infection can be induced by the use of a single antigen.

The results presented here are in agreement with the findings by Deans et al. [6], here confirmed with heterologously-expressed antigen and an adjuvant that can be used in human trials. Similar to results obtained by Deans et al. [6], solid protection was only achieved following challenge and booster vaccination. A series of three vaccinations with protein formulated in adjuvant may not yield high enough antibody and/or T-cell responses to confer protection. It is possible that exposure to live parasites followed by booster immunisation contributed to a more balanced humoral and cellular response.

The data suggest that high levels of growth inhibiting antibodies are required for protection. Moreover, *in vitro* and *in vivo* growth inhibition are correlated. Based on the correlation between parasitological measures (IVG, day to patency or day to treatment) and GIA IC_50_ values it is tempting to speculate that in this experiment growth inhibiting antibodies are the main mediators of protection. This is in agreement with the results from Dutta et al. in the *Aotus P. falciparum* model [8], the observation that passive transfer of hyperimmune IgG can abrogate parasitaemia [35] and that transplacental IgG provides protection in neonates [36], [37].

The PkAMA1 vaccinated animal that was protected during the first challenge had high GIA and IFN-γ levels. At the second and third challenge, however, IFN-γ increased in all animals (including non-protected controls) to levels similar as observed in the aforementioned animal. Thus high IFN-γ levels alone are not a correlate, although it cannot be excluded that Ag-specific IFN-γ production enhances antibody-mediated protection. Of note is that IFN-γ levels increased after challenge and decreased following booster vaccinations, whereas the inverse was observed for IL-13 levels. Overall a gradual increase in IFN-γ levels was observed for the Pk and PfAMA1 groups over the 3 challenges, suggesting improved cellular immune responses. In mice it has been demonstrated that AMA1-specific T-cells play an important role in the protection against *P. chabaudi* [38], suggesting that the improved cellular responses may have contributed to the protection observed at the second and third challenge.

Results obtained with heterologous prime-boost regimens with 4 antigens (2 pre-erythrocytic and 2 blood-stage, including AMA1) combined show that rhesus monkeys can be protected from sporozoite challenge [39], [40]. Some animals became patent, but were able to control parasitaemia, similar to what is reported here. Unexpectedly, the protected animals in the study by Weiss et al. did not have appreciable GIA levels at the time of challenge and GIA levels only developed during challenge [41], suggesting that T cells induced by vaccination provided help for antibody formation upon parasite exposure. GIA and anti AMA1 or MSP1_19_ IgG levels at the time of drug cure, however, were similar in animals that were able to control the parasitaemia and animals that were not [41]. This suggest that other, non identified, cell-mediated mechanisms may account for the protection, as IFN-γ levels at the time of challenge did not explain the ability to self cure [39]. The increase of GIA titres during challenge is in contrast with data presented here; GIA levels waned during the first and second challenges in all 6 and 4 out of 6 PkAMA1 vaccinated animals. During the third challenge 3 PkAMA1 vaccinated animals retained their titre (<5% drop) and 3 decreased. The number of animals able to retain GIA levels increased with each challenge thus it appears that boosting and parasite exposure improves immune responses in PkAMA1 vaccinated animals, possibly through the expansion of AMA1-specific T cell responses.

The results presented here indicate that high GIA levels are required for protection (IC_50_≤3.5 mg mL^−1^), but these levels wane quickly, even when exposed to parasites. This begs the question on how long protective levels can be maintained. The vaccination regimen used here closely resembles the WHO Expanded Programme on Immunisation comprising immunisations at 6, 10 and 14 weeks, and a vaccination at 9 months of age (www.who.int). Several papers indicate that immunisation timing [42], prime boost regimes or a combination of schedule and prime boost may be more effective at eliciting both cellular and antibody responses [39], [43]. It is tempting to speculate that combinations of prime with DNA or viral vectors (e.g. adenovirus, attenuated pox viruses, attenuated measles, virosomes or other virus-like particles) followed by a (heterologous) virally vectored and protein adjuvant boost would induce high humoral and cellular responses and thereby yield more solid protection. Whether such an immune response elicited by prime boost schedules is higher, persist longer and would protect upon the first challenge remains to be investigated.

The protection observed in the work reported here appears in contrast with results from a recent phase IIa study [20] where only a small, biologically non relevant, delay in pre-patent modelled-PCR data was observed despite high GIA titres to the, homologous, challenge strain. The phase IIa study involved mosquito challenge of volunteers, whereas the infection of the animals was done by intravenous passage of a low number of (viz. 10,000) iRBC. It is possible that sporozoite passage through the skin may have induced tolerance as was recently hypothesised [44]. Thus the route of infection and initial infection load may, in part, explain the differences. Moreover, human subjects have to be drug cured when positive on a thick smear, while a longer observation window (up to 2% parasitaemia) was possible for the animals, thus the control of parasitaemia as observed in the current study could not have been observed in a phase IIa setting. In any case, it will be interesting to compare mosquito challenge with blood-stage challenge in PkAMA1-vaccinated rhesus monkeys in future studies.

A recently reported phase IIb trial with AMA1 formulated in AS02 in Malian children [23] revealed a hint of protection (17%, not significant) against all *P. falciparum* infections, but significant protection (64%) when infections with parasites bearing an AMA1 homologous to the vaccine antigen were considered. Infections occurred against a background of pre-existing immunity which was boosted by PfAMA1 vaccination; a situation similar to that of the animals at the second and third challenges.

The protection observed in Malian children [23], the *Aotus* model [8] and in the current study, suggest that phase IIa studies for down-selection of vaccine candidates may need to be interpreted carefully. It would be interesting to explore whether clinically relevant protection would have been observed if the subjects in the AMA1 Phase IIa study [20] were boosted and re-challenged, as a series of vaccinations followed by infection and booster immunisation could be considered representative for the field situation.

In addition, the PkAMA1 vaccine candidate described here may also be an attractive human vaccine candidate given the fact that *Plasmodium knowlesi* is a zoonosis that affects humans and may be a contributing cause of death [13], [14].

## Supporting Information

Figure S1 **Relation between total IgG and GIA titre in PkAMA1-vaccinated animals.** Panel A: Day 70, Panel B: Day 217 and Panel C: Day 450. The amount of total IgG is depicted on the x-axis and the corresponding growth inhibition is depicted on the y-axis. The IC_50_ values are indicated by the red lines.
(EPS)
